# Therapeutic contact intensity as an adjunct to a cognitive behavioral smoking cessation app: protocol for a randomized controlled trial

**DOI:** 10.1186/s13722-026-00684-0

**Published:** 2026-06-11

**Authors:** Ana López-Durán, Carmela Martínez-Vispo, Úrsula Martínez, Rubén Rodríguez-Cano, Laura Cortés-García, M. Carmen Míguez, María Barroso-Hurtado, María Ramos-Carro, Elizabeth Moss-Alonso, Jorge Vacas, Lois Míllara, Alejandro Tubío, Elisardo Becoña

**Affiliations:** 1 Smoking and Addictive Disorders Unit, Faculty of Psychology, University of Santiago de Compostela, Santiago de Compostela, Spain; 2 Institute of Psychology (IPsiUS), University of Santiago de Compostela, Santiago de Compostela, Spain; 3 Department of Clinical Psychology and Psychobiology, Faculty of Psychology, University of Santiago de Compostela, Santiago de Compostela, Spain; 4 Department of Family and Preventive Medicine, University of Utah, Salt Lake City, UT USA; 5 Department of Psychology, Norwegian University of Science and Technology, Trondheim, Norway; 6 AT Strength and Conditioning, Vilagarcía de Arousa, Spain

**Keywords:** Cigarette smoking, Cessation, Therapeutic contact, App, Cognitive-behavioral intervention

## Abstract

**Background:**

Smoking cessation improves the health and well-being of people who smoke. It also benefits society, given the high economic and personal costs of smoking-related diseases. With the emergence of new digital technologies, there has been a shift in the delivery of smoking cessation interventions, moving from the traditional in-person treatments to remote digital interventions. In particular, despite a tremendous increase in the number of smoking cessation mobile applications, very few have been empirically tested. Additionally, it remains unclear whether therapeutic contact could improve smoking abstinence outcomes. Therefore, the overall goal of this study is to assess the efficacy of a newly developed app adapted from an in-person efficacious cognitive-behavioral treatment (CBT) for smoking cessation compared to the app in combination with different doses of therapeutic contact.

**Methods:**

We will conduct a randomized controlled trial (RCT) with a sample of 420 individuals seeking smoking cessation treatment at the Smoking and Addictive Disorders Unit of the University of Santiago de Compostela (Spain). Participants will be randomly assigned to: (1) 8 weeks of smoking cessation app (Only app; *n* = 140); (2) 8 weeks of smoking cessation app + 8 weekly sessions of group CBT delivered via video calls (High intensity; *n* = 140); or (3) 8 weeks of smoking cessation app + 4 biweekly sessions of group CBT delivered via video calls (Low intensity; *n* = 140).

**Discussion:**

We expect that the high intensity therapeutic contact group will result in higher 7-day point-prevalence smoking abstinence (validated via carbon monoxide) at the end of treatment and 3-, 6-, and 12-month post-treatment, compared to the other two conditions. This study will be the first RCT evaluating the dose of therapeutic contact needed when using a smoking cessation app. Findings will contribute to improving the efficacy and reach of smoking cessation treatments.

**Trial registration:**

ClinicalTrials.gov number: NCT06900985 registered on March 21, 2025. Protocol version 1.0.

## Background

Smoking is a well-established risk factor for both physical [1] and mental health [2], and remains the leading preventable cause of morbidity and mortality worldwide [3, 4]. In recent decades, significant advances have been made in tobacco control in most countries, including Spain. As a result, the prevalence of smoking has been decreasing progressively over time. However, smoking rates in Spain are still high, with 17% of the adult population smoking daily [5]. This results in high personal and economic costs due to the associated premature mortality and the economic burden at the health care level [6].

Smoking cessation interventions, including cognitive-behavioral approaches [7], have proven to be efficacious to quit smoking [8], but accessibility and utilization of these interventions remain a challenge [9]. Therefore, it is crucial that we develop highly efficacious treatments that can reach a broader population. In recent years, Information and Communication Technologies (ICTs) have become powerful in facilitating communication and information sharing, and, especially, for providing healthcare. The COVID-19 pandemic has further accelerated the development and adoption of these technologies, including video calls for remote interventions targeting a variety of physical (e.g., diabetes, heart failure), and psychological problems (e.g., anxiety, depression) [10, 11]. Studies have shown no differences in mental health treatment outcomes when the same intervention is conducted in person compared to a video call format [12–14]. Additionally, there has been a tremendous proliferation of mobile health (mHealth) apps to deliver a wide variety of clinical interventions [15]. Apps have numerous advantages over in-person treatments. For example, apps can remove geographic and financial barriers to treatment, increasing access to care [16]. Despite the number of advantages that mHealth interventions have, they also have a number of barriers. High rates of dropout and low treatment adherence have been reported as one of the main limitations [17].

In recent years, we have experienced a high increase in the number of smoking cessation mobile apps. However, few have been empirically tested and they have shown limited efficacy for smoking abstinence [18]. In this line, Bricker et al. [19] conducted a randomized controlled trial to test the efficacy of an app (iCanQuit) based on acceptance and commitment therapy, compared with a National Cancer Institute app (QuitGuide) based on US clinical practice guidelines. Findings from this study showed that self-reported 30-day point prevalence abstinence at 12 months post randomization was 28.2% for iCanQuit vs. 21.1% for QuitGuide.

Previous systematic review and meta-analysis suggested that adherence to digital interventions can be improved by incorporating therapeutic contact [20]. Specifically, it seems that self-guided interventions combined with health professional contact before and during treatment improved outcomes compared with no contact [21, 22]. Smoking cessation apps can be used as stand-alone interventions or as combined interventions guided by health professionals, resulting in treatments of varying intensities [23]. Therefore, different levels of intervention dose should be established, ranging from self-guided interventions (such as using Apps) to those conducted by professionals following a stepped-care approach [24, 25]. However, research examining the effect of in-person contact of varied intensity from a professional on digital interventions for substance use has been scarce. Eék et al. [26] and Sundström et al. [27] conducted a randomized controlled trial in a sample of participants with alcohol use disorder, examining the efficacy of a therapist-guided high‐intensity internet intervention (web-based) compared with an unguided low‐intensity internet intervention. No significant differences were found between conditions in the short [27] and the long term [26], suggesting that low-intensity internet interventions may be at least as efficient as high-intensity ones.

Considering the relevance of smoking cessation treatment reach and the impact of treatment adherence on cessation outcomes, examining the role of therapeutic contact in digital interventions to quit smoking is warranted. The aim of this study is to assess the efficacy of a smoking cessation app compared to the same app in combination with different doses of therapeutic contact (high vs. low) via a randomized controlled trial (RCT). Secondary aims include the assessment of treatment adherence, participant satisfaction, and potential moderators (e.g., age, education) and mediators (e.g., depression, withdrawal symptoms).

## Methods

### Study design

**Fig. 1:**
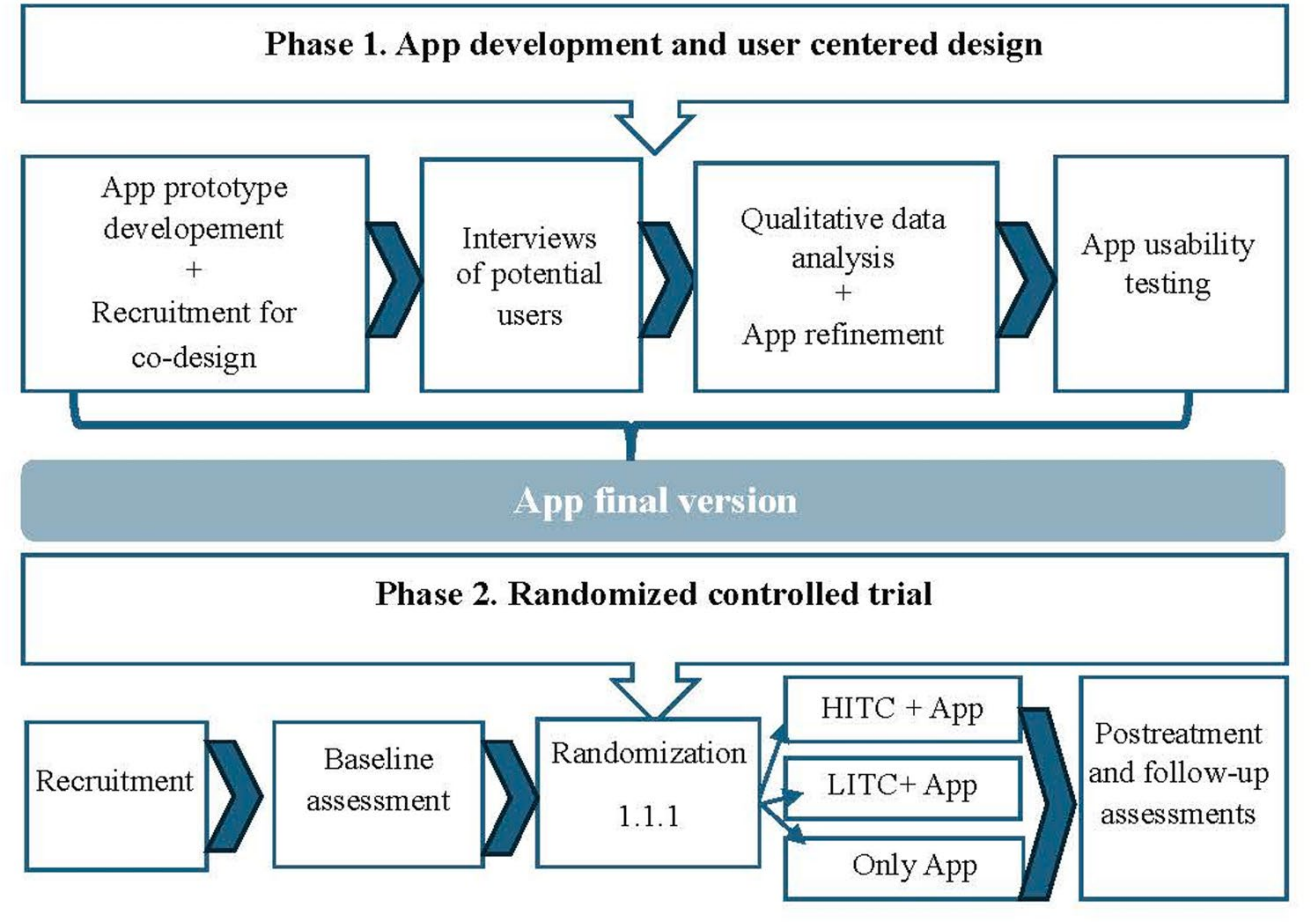
Study design overview: App development and RCT. *Note*. HITC: High Intensity Therapeutic Contact (*n* = 140); LITC: Low Intensity Therapeutic Contact (*n* = 140); Only App (*n* = 140)

The study includes two phases. The aim of the first phase is to design the app prototype by a multidisciplinary team composed of psychologists and telecommunication and computer engineers. We used a qualitative research approach, conducting in-depth individual interviews with the target population to understand their preferences for smoking cessation apps, potential barriers to use, and general feedback on how to make the app more attractive. In the second phase, we will conduct a three-arm, RCT to assess the efficacy of the app compared to different doses of therapeutic contact (high vs. low) (Fig. 1). This protocol is consistent with SPIRIT (Standard Protocol Items: Recommendations for Interventional Trials) guidelines [28].

### Participants and study procedures

Participants in both phases 1 and 2, will be individuals seeking smoking cessation treatment at the Smoking and Addictive Disorders Unit of the University of Santiago de Compostela (Spain). Recruitment will be conducted via social media, press communications, flyers, health care referrals or by word of mouth. Potential participants will contact the Unit staff by telephone or email. Prior to enrollment, they will be scheduled for an eligibility assessment session through videoconference. To be part of the study, participants will meet the following inclusion criteria: age 18 or over; interested in receiving smoking cessation treatment; provide electronic informed consent; smoking at least 5 cigarettes per day; completion of all pre-treatment assessment questionnaires; having a valid email address; having a smartphone; and being willing to use it throughout the treatment. We will exclude individuals with a diagnosis of a severe mental disorder (e.g., bipolar disorder and/or psychotic disorder) or with a concurrent substance use disorder (e.g., alcohol, cannabis); We will also exclude those reporting concurrent use of other tobacco products (electronic cigarette with nicotine, cigars or cigarillos) or who have completed an efficacious psychological or a pharmacological treatment to quit smoking during the previous year (e.g., nicotine replacement therapy). Individuals who recently had a physical pathology involving high vital risk that requires immediate intervention (e.g., recent myocardial infarction) will also be excluded. Finally, individuals with visual difficulties that prevent the use of the app will also be excluded from the study.

**Fig. 2:**
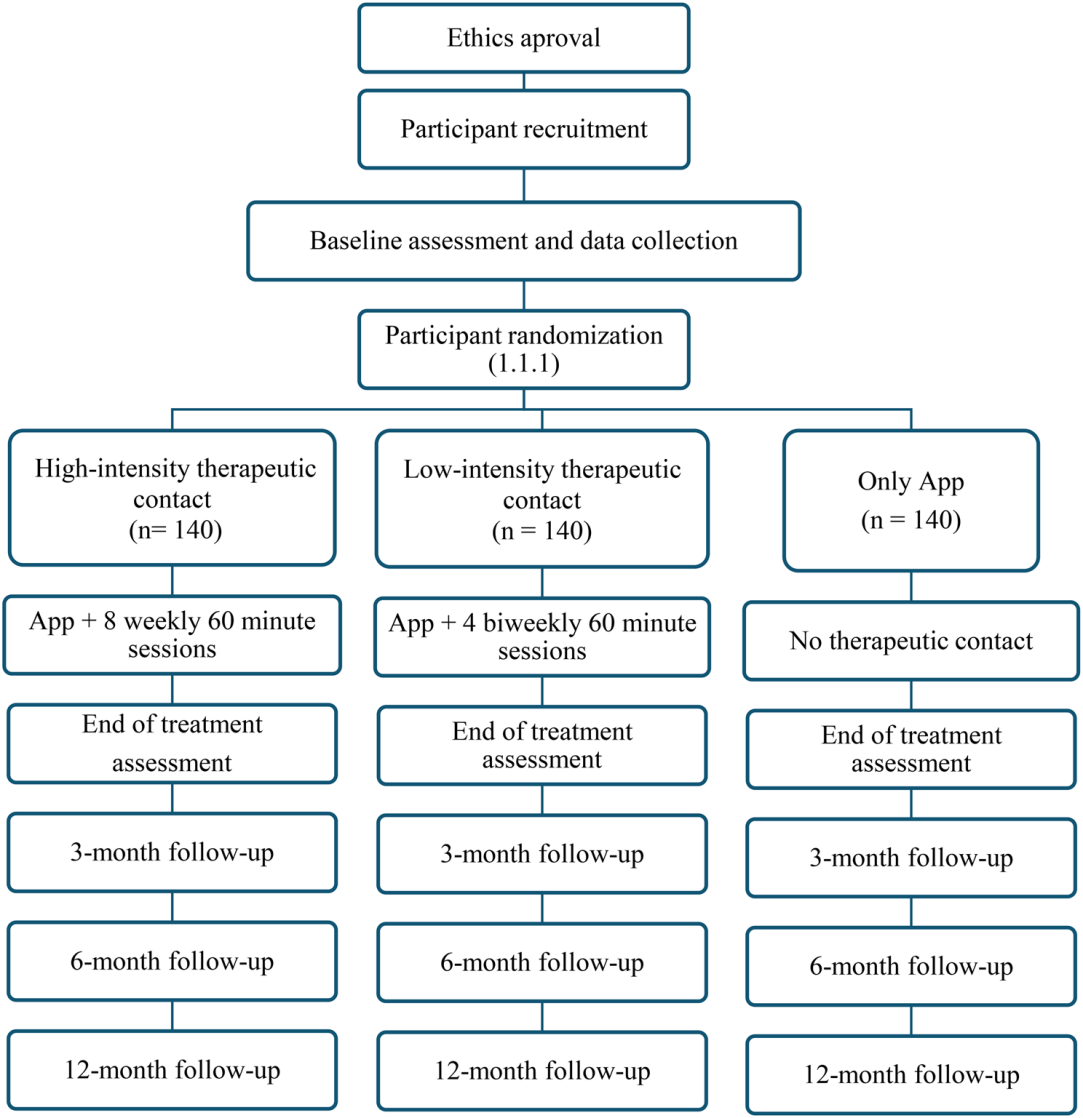
CONSORT diagram of trial progression [70]

Interested individuals meeting the inclusion criteria will then sign the electronic informed consent form. Participants in Phase 2 (RCT) will complete the baseline assessment through an individual interview, lasting approximately one hour, and via the Microsoft Teams video-calling platform. Afterwards, a link will be sent for the completion of the baseline questionnaires through Microsoft Forms. Post-treatment assessments will be conducted electronically at the end of treatment and at 3-, 6-, and 12-months follow-ups in the three treatment conditions (see Fig. 2).

### Procedures

#### Phase 1: app design

To inform the design of the app, we will conduct semi-structured in-depth individual interviews via Microsoft Teams. Before data collection, an interview guide will be developed, with key topics (e.g., previous experiences with apps and smoking cessation apps, perceived utility of apps, facilitators and barriers to using apps, app content for smoking cessation, opinions on the initial app prototype) drawn from the literature to ensure alignment with existing research. These interviews will be recorded and transcribed verbatim. Each interview will last approximately 30 to 45 min. The information collected from the interviews will be used to design and refine the app. The participants in Phase 1 will not be included in Phase 2.

Then, a beta test will be conducted to assess users’ experience with the app and determine acceptability and ease of use. For this purpose, we will recruit a convenience sample of experts (i.e., health professionals) and potential users (i.e., people who smoke). Participants in this beta test will have access to the app prototype for a period of two weeks. Then, an individual interview will be conducted by the research team to gather information that will be used to refine the app and create a final version.

#### Phase 2: randomized controlled trial

##### Randomization, allocation concealment and blinding

In phase 2, participants who meet all the eligibility criteria will be randomized to one of the three groups based on a computer-generated allocation sequence (ratio: 1.1.1). Participants will not be blinded to the treatment condition, which is common in trials evaluating psychological and psychosocial interventions due to the intervention characteristics [29].

##### Treatment conditions

Participants will be randomly assigned to one of three treatment conditions. All three conditions will have the same smoking cessation app, but they will differ in the dose of therapeutic contact (high, low, or no contact), in terms of number of sessions (8 vs. 4 vs. 0) and frequency (weekly vs. biweekly vs. no contact). Additionally, the high-intensity therapeutic contact condition involves the systematic introduction and practice of multiple behavioral and cognitive techniques by a trained therapist, whereas the low-intensity therapeutic contact condition relies more on the use of the app with the therapist providing support related to progress review, problem-solving, and reinforcement of key intervention components (e.g., smoking reduction goals).

All study participants will be followed 3-, 6-, and 12-month post-treatment.

*App content*:

Participants will have access to our smoking cessation app during their participation in the study (14 months total duration of app access). The app content is based on an evidence-based cognitive-behavioral treatment “*Programa para Dejar de Fumar*” [30, 31]. The intervention components are digitally adapted via personalization, tracking (e.g., setting physical activity goals based on baseline activity level), motivational support (e.g., having in mind that the benefits of quitting smoking are higher than the negative consequences) and achievement goals reinforcement through push notifications (e.g., You have achieved the 100% of your weekly goal!) and gamification (i.e., a badge system for abstinence goals achievement). These features are intended to increase engagement, reinforce progress, and support adherence to smoking-reduction and abstinence goals.

The program includes the following components that are delivered over the course of 8 weeks: (1) information about the app; (2) tracking the number of cigarettes smoked per day; (3) psychoeducation on tobacco and smoking; (4) nicotine fading through weekly cigarette reduction goals (30% reduction every week from first treatment week) including a personalized quit day (estimated on the fifth or sixth treatment week); (5) stimulus control by selecting daily situations in which they will progressively stop smoking (e.g., driving); (6) coping with urges to smoke and withdrawal syndrome; (7) weekly physical activity goals tailored to individuals baseline level; (8) social support; (9) anhedonia and mood-focused behavioral activation tasks; (10) smoking relapse prevention; 11) motivational notifications; and 12) a reward system for goal achievement (gamification). In addition to all the previous components, during the follow-up period participants will also have access to the following components: (1) strategies for coping with craving and motivational strategies; (2) gains and achievements after quitting; (3) tips for maintaining abstinence or quitting smoking; (4) possibility of tracking cigarette use and setting a new quit date.

Although our app shares components with other evidence-based apps, such as push notifications, badges, tracking the number of cigarettes smoked per day, or gains and achievements after quitting, it differs from existing smoking cessation apps in several app-specific features. Whereas the present app operationalizes a structured CBT smoking cessation program with evidence in Spain [32], with several digitally adapted features, particularly personalized nicotine-fading goals, individualized physical activity goals, behavioral activation exercises, among others, previous existing apps include components based on other framework, such as Acceptance and Commitment Therapy (i.e., iCanQuit) [33], mindfulness (Cravingtoquit) [34], or US clinical practice guidelines (i.e., QuitGuide; QuitSTART) [35]. Additionally, the app is designed to be used as a standalone app but also to be combined with standardized professional support.

*Treatment conditions (TC)*:

*Only App.*

Participants will have access to the smoking cessation app, with no therapeutic support (technical support will be provided if necessary).

*App + High-intensity therapeutic contact.*

Participants will receive 8 one-hour weekly group sessions via the Microsoft Teams video-calling platform, and they will have access to the app. All sessions will be conducted by clinical psychology/general health psychology professionals. The app components we previously described will be discussed in depth during the 8-week online sessions. Participants will have access to the topics discussed during session within the app.

*App + low-intensity therapeutic contact.*

Participants in this condition will receive four one-hour sessions applied in a group format every two weeks via the Microsoft Teams video-calling platform and will have access to the app. The four sessions will have the following content: (1) explanation of treatment rationale and components available in the app; (2) participants’ progress monitoring, reinforcement of achieved goals, and analysis of difficulties; (3) cessation planning support; and (4) relapse prevention support. The sessions will be conducted by clinical psychology/general health psychology professionals.

### Sample size calculation

We expect to reach theoretical saturation after interviewing 30 participants in Phase 1 to discuss app design and beta testing [36]. However, we will conduct additional interviews if new information continues to emerge.

To determine the sample size for the RCT (Phase 2), the statistical program G* power [37, 38] was used. We estimate a 20-percentage point difference in smoking abstinence between the treatment conditions with therapeutic contact at the 12-month follow-up (estimated percentages of 45% for High-intensity and 25% for Low-intensity). This estimate was based on a previous study in which the higher abstinence rate of a condition with 8 sessions and an App reached the 42.6% of 7-day PPA at 12-month follow-up [39]. Therefore, we hypothesize that 45% of participants in the High-intensity condition will be abstinent, as the intervention in the present study includes additional components compared with our previous study (i.e., tailored physical activity, social support, and anhedonia- and mood-focused behavioral activation tasks). Given that the Low-intensity condition comprises fewer therapist-guided sessions, we conservatively estimated approximately a 50% lower abstinence rate at the 12-month follow-up compared with the High-intensity condition. Therefore, assuming a 20-percentage point difference in abstinence outcomes between the High-intensity and Low-intensity conditions, a two-sided α of 0.05, and 90% power, the required sample size was 120 participants per group. We anticipate a 15% loss to follow-up based on our previous research, so we increased the per-group sample size to 140 participants. Consequently, the total sample size required for the study is 420 participants.

### Data collection

The following instruments will be used (see Table 1, which depicts the timeline for data collection).

- *Smoking Habit Questionnaire* [40]. A 59-item questionnaire assessing participant sociodemographic characteristics (e.g., sex, age, education) and smoking-related behaviors: current and past use of cigarettes and other tobacco products; age of smoking onset; previous quit attempts or cigarette reduction; reasons for quitting in previous attempts; past and current diseases; symptoms and discomforts related to smoking; alcohol, coffee/tea, and medication use; desire to quit smoking; desire to participate in the treatment; expectations about treatment; and stages of change [41].
- *The Fagerström Test for Cigarette Dependence* (FTCD) [42, 43]. Includes 6 items to assess cigarette dependence.
- *Structured Interview Assessment of DSM-5 Diagnostic Criteria for Tobacco Use Disorder*. This is a diagnostic interview developed following the DSM-5 criteria to assess tobacco use disorder.
- *Beck Depression Inventory-II* (BDI-II) [44, 45]. This is a 21-item self-report scale measuring current depressive symptoms.
- *Snaith-Hamilton Pleasure Scale* (SHAPS) [46, 47]. This 14-item scale assesses 4 domains of hedonic experience: interest/hobbies, social interaction, sensory experience, and satisfaction with food/drink.
- *Minnesota Nicotine Withdrawal Scale* (MNWS) [48]. This 8-item scale measures nicotine withdrawal symptoms (depression, insomnia, irritability/frustration/anger, anxiety, difficulty concentrating, restlessness, increased appetite/weight gain) and craving (smoking urgency).
- *Self-Efficacy/Smoking Temptation Scale* [49]. This 9-item instrument assesses the temptation to smoke in positive affect/social situations, negative affect situations, and habit situations.
- *Questionnaire of Smoking Urges* (QSU) [50]. The QSU consists of 10 items assessing craving to smoke. It has two factors: (1) the intention or desire to smoke, and (2) expectations of negative reinforcement or improvements through smoking.
- *Multidimensional Scale of Perceived Social Support* (MSPSS) [51, 52]. This 12-item self-report scale assesses perceived social support from family, friends, and others.
- *The 20-item Partner Interaction Questionnaire* (PIQ-20) [53]. This questionnaire assesses social support for adults wishing to stop smoking.
- *Environmental Reward Observation Scale* (EROS) [54, 55]. This 10-item self-report assesses the amount and availability of environmental reward.
- *Behavioral Activation for Depression Scale Short Form* (BADS-SF) [56, 57]. This scale includes 9 items assessing activation and avoidance behaviors.
- *International Physical Activity Questionnaire Short Form* (IPAQ-SF) [58]. This 7-item questionnaire measures participation in vigorous- and moderate-intensity physical activity and sedentary behaviors over the past seven days.
- *Satisfaction with Life Scale* (SWLS) [59, 60]. A scale designed to measure global life satisfaction through 5 items.
- *The visual-analogue scale of the EuroQoL-5D Quality of Life Questionnaire* (EQ-5D) [61]. It assesses health status perception on a scale ranging from 0 to 100.
- *Questionnaire about the use of smartphones and Apps*. An ad hoc questionnaire will be designed to collect information about the use of smartphones (i.e., frequency, time of use, type of use) and Apps (i.e., frequency of use, hours of use, types of Apps).
- *User Version of the Mobile Application Rating Scale* (uMARS) [62, 63]. This is a 20-item measure that includes 4 objective quality subscales (engagement, functionality, aesthetics, and information quality) and one subjective quality subscale.
- *System Usability Scale* [64, 65]. This 10-item instrument assesses the usability of a system (software interfaces, webpages, and mobile apps), alternating positive and negative statements.
- *Self-report of smoking*. Participants will track their smoking behavior by registering each cigarette, time when they smoked, the situation, and how much pleasure they experience with each cigarette on a scale from 0 to 10.
- *Assessment of Carbon monoxide in expired air (CO).* The iCOquit^®^ Smokerlyzer^®^ (Bedfont Scientific Instruments Ltd, Sittingbourne, Kent, United Kingdom) will be used to measure carbon monoxide (CO) in expired air. This is a personal device designed for the remote assessment of carbon monoxide, providing a particles per million (ppm) reading [66, 67]. Participants who self-report 7-day point-prevalence abstinence at any time-point will receive a CO monitor device via postal mail, together with detailed instructions for app installation, Bluetooth pairing, and CO measurement. Research staff will schedule a brief video call to assist and supervise the CO collection.
- *End-of-treatment questionnaire*. This self-report collects information about quit date, confidence in remaining abstinent, physical, and psychological improvements since the beginning of treatment, and possible negative effects of the intervention.
- *Client Satisfaction Questionnaire* (CSQ-8) [68, 69]. This 8-item instrument measures overall satisfaction with treatment services and will be administered at the end of treatment.
- *Follow-up questionnaire*, in which tobacco related information is collected at 3-, 6-, and 12- month post-treatment.

**Table 1:** Timeline for data collection through the trial

		STUDY PERIOD				
	Enrollment	Allocation	EOT	3-M	6-M	12-M
**ENROLLMENT**						
Eligibility screen	X					
Informed consent	X					
Allocation		X				
**INTERVENTIONS**						
TC 1 High intensity contact						
TC 2 Low intensity contact						
TC 3 Only app						
**ASSESSMENTS**						
Smoking Habit Questionnaire		X				
DSM-5 Diagnostic Criteria for TUD		X				
FTCD		X				
BDI-II		X	X	X	X	X
SHAPS		X	X	X	X	X
MNWS		X	X	X	X	X
Self-Efficacy/Temptation Scale		X	X	X	X	X
QSU		X	X	X	X	X
MSPSS		X	X	X	X	X
PIQ-20		X	X	X	X	X
EROS		X	X	X	X	X
BADS Short Form		X	X	X	X	X
IPAQ-SF		X	X	X	X	X
SWLS		X	X	X	X	X
EuroQoL-5D		X	X	X	X	X
Use of smartphones and Apps Questionnaire		X				
CSQ-8			X			
SUS			X			
uMARS			X			X
***Primary outcome***						
7-days point prevalence abstinence						X
***Secondary outcomes***						
CSQ-8			X			
7-days point prevalence abstinence			X	X	X	

TC = Treatment Condition; EOT = End of treatment; 3-M = 3 months follow-up; 6-M = 6 months follow-up; 12-M = 12 months follow-up; TUD = Tobacco Use Disorder; FTCD = Fagerström Test for Cigarette Dependence; BDI-II = Beck Depression Inventory-II; SHAPS = Snaith-Hamilton Pleasure Scale; MNWS = Minnesota Nicotine Withdrawal Scale; QSU = Questionnaire of Smoking Urges; MSPSS = Multidimensional Scale of Perceived Social Support; PIQ-20 = Partner Interaction Questionnaire; EROS = Environmental Reward Observation Scale; BADS-SF = Behavioral Activation for Depression Scale Short Form; IPAQ-SF = International Physical Activity Questionnaire Short Form; SWLS = Satisfaction with Life Scale; EQ-5D = EuroQoL-5D Quality of Life Questionnaire; SUS = System Usability Scale; uMARS = User Version of the Mobile Application Rating Scale; CSQ-8 = Client Satisfaction Questionnaire

#### Primary outcome

Smoking abstinence in the three study conditions will be self-reported and biochemically validated using carbon monoxide (CO) in exhaled air [71], using the iCOquit^®^ Smokerlyzer^®^. These devices will be mailed to participants. A participant will be classified as abstinent from smoking if they report not smoking, not even a puff, in the previous 7 days and provide a CO reading < 6 ppm at 12 months [72]. Following an intent-to-treat approach, participants lost to follow-up will be considered as smoking.

#### Secondary outcomes

- 7-day point prevalence smoking abstinence at 7-days post-quit day, and at 3- and 6-months post-intervention.
- Treatment satisfaction, measured with the CSQ-8 [68, 69] and the SUS [64, 65], at the end of treatment.
- Treatment adherence. It will be assessed to evaluate the extent to which participants use the intervention. Measures of adherence will include:
  - Completion of intervention components and the number of uses within each component will be obtained from the app metadata.
  - Data obtained from the engagement subscale of the uMARS [62, 63].

For conditions with therapeutic contact, adherence will also be assessed based on the number of online sessions participants attend.

### Statistical analysis

#### Phase 1: app design

Interviews will be recorded, transcribed verbatim, and independently coded using NVivo 14 [73]. We will first develop a codebook. Initial codes will be guided by the interview guide and the original research objectives. Then, these codes will be refined, operationalized, and defined based on the raw interview data. The interviews will be coded independently by two researchers, and then iteratively revised until an intercoder reliability coefficient of at least 0.80 is achieved. Discrepancies will be addressed through continued discussion with a third researcher when needed [74, 75]. After coding is completed, we will conduct a thematic analysis to identify emerging themes.

#### Phase 2: RCT

All statistical analysis will be conducted following an intention-to-treat approach [76] in which all randomized participants will be included in the final analysis. Sociodemographic (e.g., age, sex, gender, education), tobacco-related and clinical characteristics will be reported via descriptive statistics. Parametric (χ^2^ and Student’s t) and non-parametric test (Mann-Whitney U) will be used to examine baseline differences between treatment conditions in sociodemographic variables, tobacco use, clinical variables and variables related to the use of mobile phones and apps. Given that our treatment conditions that include therapeutic contact are delivered in a group format, the primary analysis will account for clustering by treatment/contact group. The pattern of missing data will be analyzed, and multiple imputation techniques will be used based on the missingness pattern.

To analyze differences between treatment conditions, percentages of smoking abstinence (χ^2^) and effect size calculation (Cramer’s V) will be used. Due to the longitudinal nature of the collected data, statistical analyses regarding smoking abstinence outcomes will be conducted through multilevel modeling.

Moderation analysis will be conducted to examine whether treatment effects differ as a function of participant baseline characteristics, such as nicotine dependence, age, or education level. These analyses will test whether the intervention has different abstinence outcomes for specific subgroups by including treatment-condition × baseline characteristic interaction terms in the longitudinal multilevel models. We will also explore which variables could act as mechanisms of change via mediation models. Mediators will include negative affect, withdrawal symptoms, and self-efficacy.

### Ethics approvals and dissemination

The study protocol, informed consent forms, and recruitment materials have been approved by the Bioethics Committee of the University of Santiago de Compostela (USC10/2025). The study will be conducted, and results will be presented in conformity with the Consolidated Standards of Reporting Trials (CONSORT) guidelines [70] and CONSORT-EHEALTH checklist [77]. Signed informed consent will be obtained from all the participants electronically. This randomized controlled trial was registered on March 21, 2025, on ClinicalTrials.gov (NCT06900985).

The results will also be disseminated to society, especially to people interested in quitting smoking, through our website (https://www.usc.gal/gl/servizos/area/seguridade-saude), social media networks (@UTTA.USC; @unidade_tabaquismo_USC; @universidade_USC), and mass media (radio, TV, written press). We also plan to disseminate the obtained results to healthcare professionals.

### Potential risks and safety

It is not expected that participants in the trial will experience harm. However, during the smoking cessation treatment, participants may experience smoking withdrawal symptoms for several weeks. Clinical psychologists and other staff at the Smoking Cessation and Addictive Disorders Unit will be available in case participants experience severe physical or mental symptoms related to the research study. All serious symptoms will be recorded and reported.

### Confidentiality and data management plan

The management of study data will adhere strictly to the provisions set forth in Regulation (EU) 2016/679 of the European Parliament and of the Council of 27 April 2016 (General Data Protection Regulation), concerning the protection of natural persons in relation to the processing of personal data and the free movement of such data. Additionally, compliance with relevant Spanish legislation will be ensured, including Organic Law 3/2018 of 5 December on the Protection of Personal Data and the Guarantee of Digital Rights, as well as Law 41/2002 of 14 November, which establishes the basic legal framework for patient autonomy and rights and obligations in the field of clinical information and documentation.

Participant data will be collected electronically using a unique identification code assigned to each individual and will be stored in password-protected folders on computers at the Smoking Cessation and Addictive Disorders Unit, University of Santiago de Compostela (Spain). All study-related data will be securely exported and stored under these coded identifiers to maintain anonymity, with personal identifiers, such as eligibility screening data and informed consent forms, kept separately from the main study records. Access to the data will be restricted to the principal investigator and authorized research staff only, ensuring strict confidentiality and data integrity throughout the trial. Individual de-identified participant data, data dictionary and statistical code will be accessible in Zenodo repository.

A data monitoring committee is not required for this research. The intervention involves minimal risks, and internal safety monitoring is included in the study procedures.

## Discussion

This study protocol presents the design of a three-arm RCT that aims to assess therapeutic treatment dose as an adjunct to a cognitive-behavioral smoking cessation treatment with an App. The study also includes an initial qualitative phase focused on user-centered app design, development, and testing to ensure the app is appealing and engaging [23].

Previous research suggests that professional support enhances adherence to digital interventions for substance use [20], thereby improving treatment outcomes. Therefore, it is hypothesized that participants in the high-intensity therapeutic contact condition will show significantly higher abstinence rates at the end of the intervention and across the one-year follow-up period compared to those receiving the low therapeutic contact condition or only the app. Despite expectations that smoking abstinence outcomes would be superior in the high-intensity contact condition, the app-only condition should be considered for its potential for broad dissemination. Self-guided mHealth interventions could be viable options because they are cost-efficient and may be especially appealing for people who smoke and are reluctant to seek traditional care or face difficulties attending therapist-guided sessions [16, 78]. In contrast, the therapist-guided conditions may be particularly necessary for individuals who experience specific difficulties quitting smoking, such as those with high nicotine dependence or with mental health conditions. We will explore possible moderators of treatment outcomes, that would allow for the identification of which individuals are most likely to benefit from each level of therapeutic contact. Finally, we will test the potential mediators to identify mechanisms by which the intervention leads to smoking cessation outcomes.

Despite its strengths, several limitations should be acknowledged. First, while the sample size is appropriate for Phase 1, it may not fully capture the diversity of user needs. Second, since participants are seeking treatment for smoking cessation, they may already have a higher motivation to quit smoking. This limits the generalizability of the results to the general population of people who smoke. Third, while the study will use CO biochemical verification to validate smoking abstinence, remote delivery has barriers, such as difficulties managing the device or downloading an additional app [79, 80]. However, to minimize the burden, we will schedule a brief video call not only to objectively assess abstinence, but also to assist participants with CO testing and to reduce the impact of these limitations. Fourth, study participants and research staff delivering the intervention will not be blinded to the treatment conditions. Although this is common in psychological studies, it may introduce social desirability and expectation biases. Finally, our trial is focused on abstinence outcomes and does not address whether the incremental cost and complexity of adding therapeutic contact provide a meaningful benefit that justifies the added costs, therapist time, and implementation complexity required to deliver it.

In conclusion, this study will contribute to the growing and rapidly evolving literature on digital health and smoking cessation. It will offer evidence on the need and dose of therapeutic contact as an adjunct to an app. Importantly, this trial may help shape efficacious, accessible, and scalable interventions to quit smoking. The study may also help clarify which intervention formats are most appropriate for different user groups, including those who may benefit sufficiently from a standalone app and those who may require additional therapeutic guidance. This is particularly relevant in real-world healthcare settings, where smoking cessation support should be tailored to users’ needs, preferences, and levels of digital literacy. Future research incorporating cost-effectiveness and implementation outcomes is warranted to determine whether lower-therapeutic contact or app-only interventions may represent viable options in healthcare settings. This could ultimately inform clinical guidelines or health policy decisions.

## Data Availability

No datasets were generated or analysed during the current study.
